# Skewed distribution of leaf color RGB model and application of skewed parameters in leaf color description model

**DOI:** 10.1186/s13007-020-0561-2

**Published:** 2020-02-26

**Authors:** Zhengmeng Chen, Fuzheng Wang, Pei Zhang, Chendan Ke, Yan Zhu, Weixing Cao, Haidong Jiang

**Affiliations:** 1 Key Laboratory of Crop Physiology and Ecology in Southern China, Ministry of Agriculture, Jiangsu Collaborative Innovation Center for Modern Crop Prodution, National Engineering and Technology Center for Information Agriculture, Nanjing Agricultural University, Nanjing, 210095 People’s Republic of China; 2 Jiangsu Meteorological Bureau, Nanjing, 210008 People’s Republic of China; 3 Qin Gengren Modern Agricultural Science and Technology Development (Huai’an) Co Ltd., 223001 Huai’an, People’s Republic of China; 4 Fujian Haisheng Cultural Media Co., Ltd., Fuzhou, 350003 People’s Republic of China

**Keywords:** RGB model, Leaf color, Skewed distribution, Skewed parameters, SPAD

## Abstract

**Background:**

Image processing techniques have been widely used in the analysis of leaf characteristics. Earlier techniques for processing digital RGB color images of plant leaves had several drawbacks, such as inadequate de-noising, and adopting normal-probability statistical estimation models which have few parameters and limited applicability.

**Results:**

We confirmed the skewness distribution characteristics of the red, green, blue and grayscale channels of the images of tobacco leaves. Twenty skewed-distribution parameters were computed including the mean, median, mode, skewness, and kurtosis. We used the mean parameter to establish a stepwise regression model that is similar to earlier models. Other models based on the median and the skewness parameters led to accurate RGB-based description and prediction, as well as better fitting of the SPAD value. More parameters improved the accuracy of RGB model description and prediction, and extended its application range. Indeed, the skewed-distribution parameters can describe changes of the leaf color depth and homogeneity.

**Conclusions:**

The color histogram of the blade images follows a skewed distribution, whose parameters greatly enrich the RGB model and can describe changes in leaf color depth and homogeneity.

## Background

In recent years, high-throughput techniques for phenotype identification in greenhouses and fields have been proposed in combination with non-invasive imaging, spectroscopy, robotics, high-performance computing and other new technologies, to achieve higher resolution, accuracy and fast [1, 2]. With the increasing maturity of digital image technology and the rising popularity of high-resolution camera equipment, research is becoming more feasible on qualitative and quantitative descriptions of phenotypic traits of plant appearance using digital imaging techniques [3–6]. Digital cameras can record spectral leaf information in visible color bands, with high resolutions and low costs [7]. In addition, digital color images contain rich information of plant morphology, structure, and leaf colors. So, leaf digital images are often exploited to identify changes in leaf color [8–10].

The most commonly used color representation for digital color images is the RGB color model. For an RGB color image, three color sensors per pixel can be used to capture the intensity of light in the red, green, and blue channels, respectively [11]. Existing software tools, such as MATLAB is used to process the obtained digital pictures [12]. The study of RGB color models of plant leaves has a long history [13]. After decades of development, the RGB color information of plant leaves has been exploited for the determination of chlorophyll content and indicators of changes in this content [14]. To exploit the data further, researchers suggested a number of RGB-based color features for the determination of chlorophyll levels in potato, rice, wheat, broccoli, cabbage, barley, tomatoes, quinoa and amaranth [15–23]. Many formulas have also been suggested to determine leaf chlorophyll content based on RGB components such as (R_Mean_ − B_Mean_)/(R_Mean_ + B_Mean_),G_Mean_/(R_Mean_ + G_Mean_ + B_Mean_), R_Mean_/(R_Mean_ + G_Mean_ + B_Mean_), G_Mean_/R_Mean_, R_Mean_ + G_Mean_ + B_Mean_, R_Mean_-B_Mean_, R_Mean_ + B_Mean_, R_Mean_ + G_Mean_, log sig ((G_Mean_ − R_Mean_/3 − B_Mean_/3)/255) [20]. However the problem of the small amount of information still persists. This information scarcity has become a bottleneck in the application of RGB models, greatly limiting their use.

In the analysis of RGB data of leaf images, the cumulative frequency distributions of the R_Mean_, G_Mean_ and B_Mean_ components have been generally assumed to follow a normal distribution. However, recent studies have reported that the cumulative frequency distributions of leaf colors follow skewed distributions. For example, Wu et al. found that the cumulative frequency of tea leaf color has a skewed distribution, and that the deviations with new and old leaves had clear differences [21]. Also, the moisture condition in maize leaves is related to the deviation of the grayscale values in the RGB blade model [22]. The asymmetry of a skewed distribution can be described by the partial frequency distributions of the skewed distribution curve. Several parameters can be derived from a skewed distribution including the mean, median, mode, skewness, kurtosis, and others.

The SPAD leaf chlorophyll meter is one of the most widely used hand-held meters for rapid and non-destructive assessment of the chlorophyll content in many crops [23]. In this paper, we analyzed the frequency distributions of the red, green, blue and grayscale channels in RGB leaf images and confirmed the skewed characteristics of these distributions. By extracting relevant distribution parameters, models are established for the correlation of the color characteristic parameters and the SPAD chlorophyll concentration values. When the skewness parameter was exploited, we found that both the fitting degree and the prediction accuracy were greatly improved. The proposed spatial model could predict the SPAD values more accurately, and explain the physiological significance of the leaf color changes. We hope that this work would provide researchers with a new method for the analysis of blade color patterns in RGB digital images.

## Materials and Methods

### Experimental design

In this work, the tobacco was planted in pots on November 25, 2017 at Shanghang County Township, Fujian, China (24°57′N,116°30′E). The 50-day-old seedlings were transferred to the field. Then, tags were made for 400 new tobacco leaves which exhibited consistent normal growth and leaf color, as well as no signs of pests and diseases after 15 days. A total of 100 leaves were collected at 40, 50, 60 and 65 days of leaf age, respectively. For each leaf, the SPAD value was measured at 10 AM. Then, the leaves were picked and sent to a dark room to take photos for them immediately.

### Leaf image collection

On the same day of plant sampling, tobacco leaves were transferred to one platform in a dark room. The platform used for image acquisition is a rectangular desktop of a 300-cm length, a 200-cm width, and an 80-cm height. The desktop bottom plate is a white matte scrub countertop. Images were captured using a high-resolution camera (CANON EOS-550D, Canon Company, Japan) with a resolution of 3840 × 5120 pixels. The camera was mounted on atripod at the nadir position with a constant height of 1 m above the top of the platform. The light sources are two 20-W strip white LED lamps with a color temperature of 4000 K. To ensure light uniformity, the lamp suspension positions in the platform are at 1/4th, and 3/4th of the 200 cm distance to the fixed digital camera.

### Leaf image segmentation, denoising and color feature extraction

The commercial image-editing software, *Adobe Photoshop CS*, was used to manually cut each original image, save the PNG image as a transparent background, and adjust the image size to 1000 × 1330. The *MATLAB 2016R* computing environment was used for the extraction and analysis of the color image data. First, the *imread* and *rgb2gray* functions were respectively used to read each color image and obtain its gray-level information. Then, the *double* function was used to convert each gray-level array into a double-precision array. The *mean*, *median*, *mode*, *skewness* and *kurtosis* functions were respectively used to analyze and obtain the mean, median, mode, skewness, kurtosis, and other parameters of the double-precision arrays of the red, green and blue channels as well as the gray-level image for each color leaf image.

### Color cumulative histogram construction and normality testing

The *imread* and *rgb2gray* functions are used to read each color image and obtain its gray-level counterpart. Then, using the image histogram functions, the cumulative histograms of the double-precision arrays of the red, green, blue and gray-level data were obtained. The Lilliefors and Jarque–Bera tests were used to test the distribution normality.

### Chlorophyll concentration measurement

For measuring the chlorophyll concentration, a chlorophyll meter (SPAD-502, Zhejiang Topuiunnong Technology Co., Ltd., China) was used to obtain the SPAD values for 50 pieces of fully-expanded tobacco leaves at 40, 50, 60 and 65 days of age, respectively. Each leaf blade was measured at five points: one on the upper part, two at the middle part, and two at the petiole of both sides of the leaf. The measurement process was designed to ensure that the sample completely covers the receiving window, avoid the veins only, and determine the leaf meat tissue. For each blade, the SPAD value is the mean value of the 5 measured points.

### Model building and goodness-of-fit testing

We mainly used the IBM *SPSS Statistics22* software to analyze the blade features at ages of 40, 50, 60 and 65 days, and establish multivariate linear regression models, F_1_ and F_2_, by stepwise regression. In the F_1_ model, we got the parameters (R_Mean_, G_Mean_, B_Mean_) using the mean function for three color channels. Then, we used each of these three parameters and ten combinations of them (namely (R_Mean_ + G_Mean_ + B_Mean_), R_Mean_/(R_Mean_ + G_Mean_ + B_Mean_), G_Mean_/(R _Mean_ + G_Mean_ + B_Mean_), B_Mean_/(R_Mean_ + G_Mean_ + B_Mean_), R_Mean_ − B_Mean_, R_Mean_ − G_Mean_, G_Mean_ − B_Mean_, R_Mean_ + B_Mean_, R_Mean_ + G_Mean_, B_Mean_ + G_Mean_) to establish a multivariate linear regression model by stepwise regression. The parameter equation with the highest prediction accuracy was used to construct the F_1_ model. Similarly, all 20 parameters (namely R_Mean_, R_Median_, R_Mode_, R_Skewness_, R_Kurtosis_, G_Mean_, G_Median_, G_Mode_, G_Skewness_, G_Kurtosi_, B_Mean_, B_Median_, B_Mode_, B_Skewness_, B_Kurtosis_, Y_Mean_, Y_Median_, Y_Mode_, Y_Skewness_ and Y_Kurtosis_) were used to establish a multivariate linear regression model by stepwise regression. The parameter associated with the highest prediction accuracy was used to construct the F_2_ model. Using the MATLAB software, the data was fit with Fourier and spatial functions based on all 20 parameters of 40, 50, 60 and 65 days of blade age, to establish two multivariate linear regressionmodelsF_3_ and F_4_. Then, goodness-of-fit testing was performed.

### Computer equipment

In this work, images and data were processed using a virtual private server. The hardware resources included Intel Xeon CPU E5-2640 2.5 GHz with 2 DDR4 8 GB RAMs. This server type can perform billion double-precision real-time floating-point operations.

## Results

### Distribution characteristics and normality verification of color gradation cumulative frequency of leaf-color RGB model

**Fig. 1:**
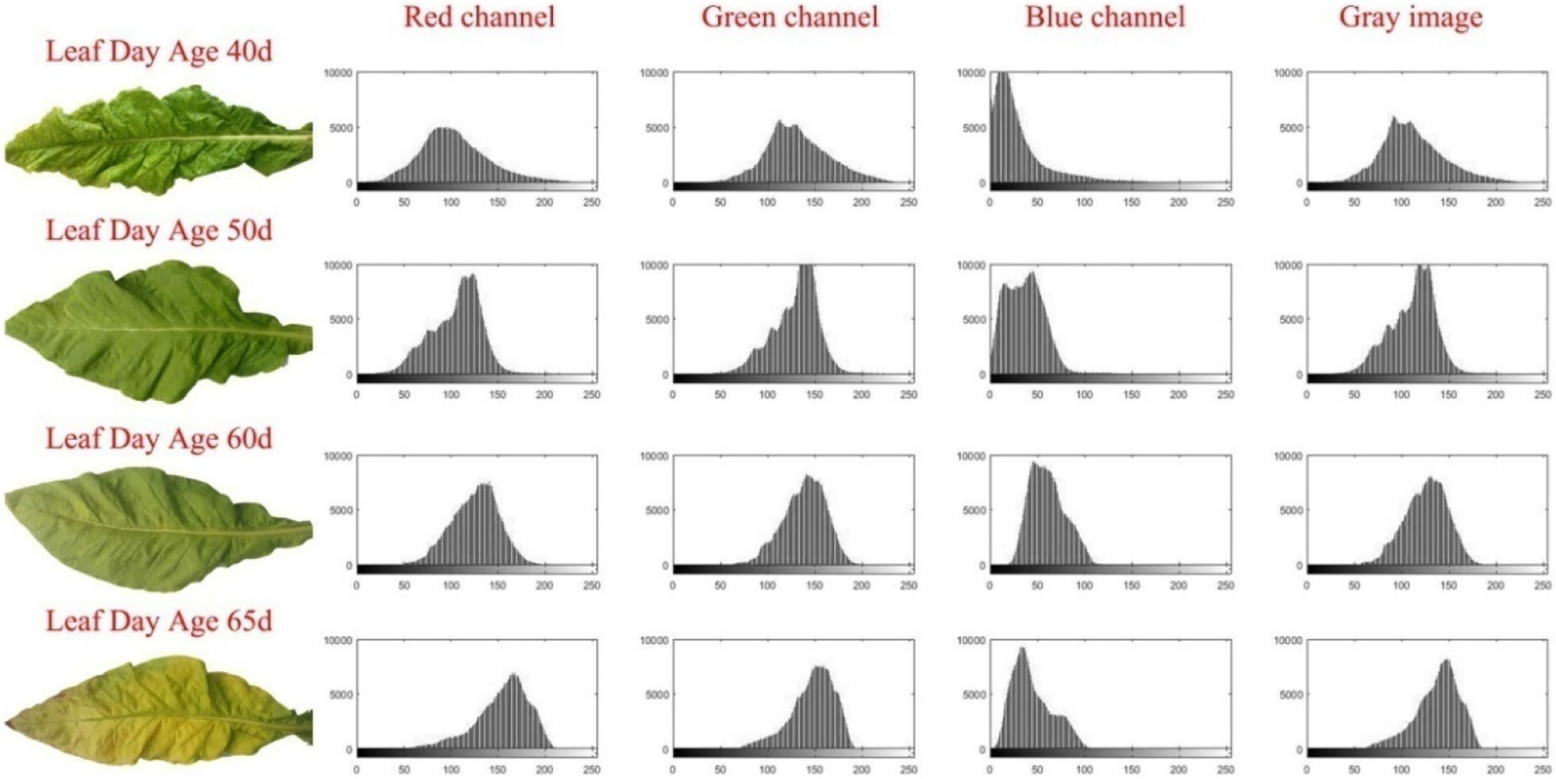
Color gradation cumulative frequency histograms for single-leaves at four different leaf ages. The leaves are picked at random. Color gradation cumulative frequency histograms of the red, green, and blue color channels as well as gray-level images are showed at 40, 50, 60 and 65 days of leaf age. The X-axis is the cumulative frequency, and the Y-axis is the intensity level frequency

In previous studies, the histogram of RGB leaf colors was mostly assumed to follow a normal distribution [24–27]. However, the validity of this assumption was contested by some reports. To verify the suitability of the proposed method, we designed an experiment that involves tobacco leaf images with different sample sizes and growth periods. We found that the tobacco leaves gradually decayed, and that the leaf color changed from green to yellow after 40 days. All histograms of single-leaf RGB images at different leaf ages (40, 50, 60, and 65 days) had skewed distributions (Fig. 1). No one RGB color distribution (red, green, blue or grayscale) was completely normal and the skewness changed regularly with the increase in the leaf age. To further confirm our histogram-based findings, we performed the Lilliefors and Jarque–Bera normality test using color gradation data of 50 leaves. The results showed that the normal distribution hypothesis value was1, and the *p* value was 0.001 (< 0.05). That means the leaf color distribution follows a skewed distribution, not a normal one.

### Correlation between skewed-distribution parameters and SPAD values

We have shown that the leaf RGB color distribution is a skewed distribution. Using skewed-distribution analysis in MATLAB, we got 20 parameters including the mean, median, mode, skewness and kurtosis for the red, green, blue and grayscale channels, respectively. In the individual-leaf color distribution, the parameters of the skewness and kurtosis represent the state of the leaf color distribution (Table 1). The skewness showed obvious changes with different leaf ages and decreased from positive to negative values. This also indicates that the color distribution of tobacco leaves is skewed throughout their lifetime. The SPAD values showed increasing and then decreasing trends.

**Table 1:** Parameters using skewed-distribution analysis and the SPAD values

Parameter	40 days	50 days	60 days	65 days
SPAD	25.02b	32.45c	25.56b	10.95a
R_Mean_	98.64a	102.38b	121.78c	154.62d
R_Median_	94.96a	104.88b	123.68c	158.68d
R_Mode_	88.32a	118.02b	131.64c	170.62d
R_Skewness_	0.46d	− 0.04c	− 0.19b	− 0.59a
R_Kurtosis_	0.14b	0.26b	− 0.07a	0.12b
G_Mean_	126.58a	126.98a	138.96b	149.80c
G_Median_	123.02a	130.26b	141.30c	153.14d
G_Mode_	114.72a	137.50b	146.60c	163.82d
G_Skewness_	0.35d	− 0.29c	− 0.36b	− 0.59a
G_Kurtosis_	0.37b	0.43b	0.06a	0.51b
B_Mean_	32.60a	37.34b	39.65b	44.82c
B_Median_	21.92a	34.48b	36.54b	41.74c
B_Mode_	12.32a	24.82b	27.14b	34.28c
B_Skewness_	1.83b	1.48a	1.65ab	2.10c
B_Kurtosis_	3.70a	7.13b	8.85b	13.32c
Y_Mean_	107.47a	109.38a	122.49b	139.28c
Y_Median_	103.14a	111.88b	124.16c	142.30d
Y_Mode_	95.08a	118.38b	127.96c	152.66d
Y_Skewness_	0.54d	− 0.09c	− 0.20b	− 0.44a
Y_Kurtosis_	0.39b	0.42b	0.05a	0.44b

The 20 parameters include the mean, median, mode, skewness and kurtosis with the red, green, and blue color channels as well as the gray-level images with MATLAB using 50 pieces of fully expanded tobacco leaves at 40, 50, 60 and 65 days, respectively. The SPAD values also come from the 50 leaves for each leaf age. Each leaf blade was measured at five points: one on the upper part, two at the middle part and two at petiole of both leaf sides. Values without a common letter are significantly different according to the Duncan test (*p *< 0.05)

We performed correlation analysis using the mean parameters (R_Mean_, G_Mean_, B_Mean_) and their combinations (namely (R_Mean_ + G_Mean_ + B_Mean_), R_Mean_/(R_Mean_ + G_Mean_ + B_Mean_), G_Mean_/(R _Mean_ + G_Mean_ + B_Mean_), B_Mean_/(R_Mean_ + G_Mean_ + B_Mean_), R_Mean_ − B_Mean_, R_Mean_ − G_Mean_, G_Mean_ − B_Mean_, R_Mean_ + B_Mean_, R_Mean_ + G_Mean_, B_Mean_ + G_Mean_) while earlier studies only used the parameters in Table 2. In Table 3, we carried on correlation analysis using 20 RGB skewed-distribution parameters with 200 leaves of four leaf ages. The results showed 17 out of 20 parameters were significantly correlated with the SPAD values at the 0.01 level. This means the change of the chlorophyll content was highly correlated with the change of the leaf color. While the chlorophyll distribution area is not uniform, it is numerically related to the increase in skewness.

**Table 2:** Correlation between the mean parameters and their combinations for tobacco leaves and the blade SPAD values

	R_Mean_	G_Mean_	B_Mean_	R_Mean_ + G_Mean_ + B_Mean_	R_Mean_/R_Mean_ + G_Mean_ + B_Mean_	G_Mean_/R_Mean_ + G_Mean_ + B_Mean_	B_Mean_/R_Mean_ + G_Mean_ + B_Mean_
Pearson correlation	− 0.763**	− 0.711**	− 0.402**	− 0.737**	− 0.723**	0.675**	0.150*

	R_Mean_ − G_Mean_	R_Mean_ − B_Mean_	G_Mean_ − B_Mean_	R_Mean_ + G_Mean_	R_Mean_ + B_Mean_	G_Mean_ + B_Mean_	
Pearson correlation	− 0.750**	− 0.743**	− 0.545**	− 0.755**	− 0.735**	− 0.650**	

The mean parameters of the red, green, and blue color channels as well as the gray-level images were obtained using 50 pieces of fully expanded tobacco leaves at 40, 50, 60 and 65 days, respectively. The SPAD values also come from 50 leaves at each leaf age. Each leaf blade was measured at the same five points mentioned in Table 2

** Indicates significant correlation according to a two-tailed test (*p *< 0.01)

* Indicates significant correlation according to a two-tailed test (*p *< 0.05)

**Table 3:** Correlation between the skewed-distribution parameters and the blade SPAD values of the tobacco leaves

	R_Mean_	R_Median_	R_Mode_	R_Skewness_	R_Kurtosis_	G_Mean_	G_Median_	G_Mode_	R_Skewness_	R_Kurtosis_
Pearson correlation	− 0.763**	− 0.728**	− 0.592**	− 0.458**	− 0.007**	0.711**	0.637**	− 0.480**	0.312**	− 0.109

	B_Mean_	B_Median_	B_Mode_	B_Skewness_	B_Kurtosis_	Y_Mean_	Y_Median_	Y_Mode_	Y_Skewness_	Y_Kurtosis_
Pearson Correlation	− 0.402**	− 0.337**	− 0.341**	− 0.268**	− 0.300**	− 0.744**	− 0.680**	− 0.526**	0.339**	− 0.078

The 20 parameters with the red, green, and blue color channels as well as the gray-level images were obtained with MATLAB using 50 pieces of fully expanded tobacco leaves at 40, 50, 60 and 65 days, respectively

** Indicates significant correlation according to a two-tailed test (*p *< 0.01)

* Indicates significant correlation according to a two-tailed test (*p *< 0.05)

### Construction of the correlation models between the SPAD and leaf color parameters

**Fig. 2:**
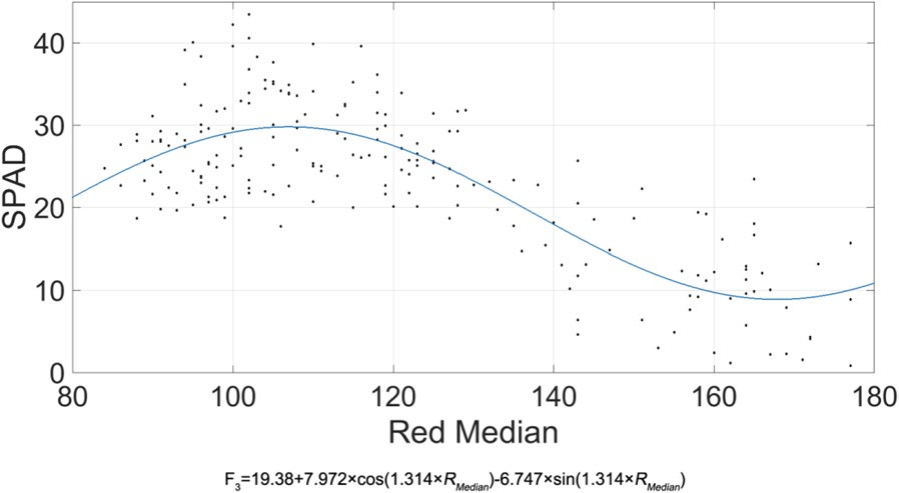
SPAD Fourier-based nonlinear fitting model. The fitting curve (F_3_)was obtained by the MATLAB Curve Fitting Toolbox

**Fig. 3:**
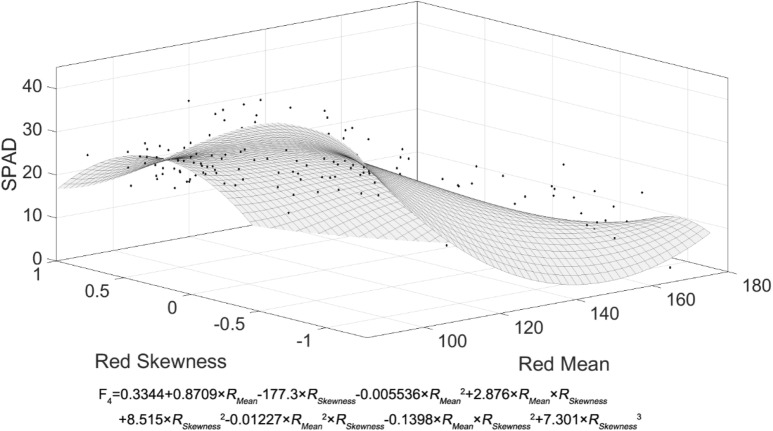
SPAD polynomial fitting surface. The fitting curve (F_4_)was obtained by the MATLAB Curve Fitting Toolbox

The correlation model can be established by the leaf color parameters based on the skewed distribution and the SPAD value. In previous studies, researchers generally used stepwise regression methods based on ordinary least squares (OLS) to construct the association model. For comparison with previous models, we used the mean parameters R_Mean_, G_Mean_, B_Mean_ and their combinations to establish multivariate linear regression models by stepwise regression, then chose the best combination as the model F_1_ (Table 4). We also extended the parameter range and adopted 20 parameters to establish multivariate linear regression models by stepwise regression, then chose the best as the model F_2_. We found that the leaf color parameters changed linearly with increasing leaf ages, while the SPAD value was characterized by first increasing and then decreasing. Since different color gradations represent different wavelengths of light, we were inspired to use the Fourier functions to fit and get the model F_3_ (Fig. 2). The leaf color showed different kinds of change, both in depth and in heterogeneity at different positions, with non-planar characteristics. Therefore, to model the bidirectional changes of leaf color (i.e. the change of leaf color depth and distribution), we used the MATLAB Curve Fitting Toolbox to fit the polynomial F4 that incorporates spatial bidirectional patterns (Fig. 3).

**Table 4:** Constructed correlation models between the SPAD value and the leaf color parameters

Model	Fit type
F_1=_59.733 − 0.304 × R_Mean_	Linear regression
F_2_ = 76.134 − 0.441 × R_Mean_ − 11.203 × *Y*_*Skewness*_ − *1.516* × G_Kurtosis_	Linear regression
F_3_ = 19.38 + 7.972 × cos (1.314 × *R*_*Median*_) − 6.747 × sin (1.314 × *R*_*Median*_)	Fourier fitting
F_4_ = 0.3344 + 0.8709 × R_Mean_ − 1 77.3 × *R*_*Skewness*_ − 0.005536 × R_Mean_^2^ +2.8 76 × R_Mean_ × *R*_*Skewness*_ + 8.515 × *R*_*Skewness*_^*2*^ − 0.0122 7 × R_Mean_^*2*^ × *R*_*Skewness*_ − 0.1398 × R_Mean_ × *R*_*Skewness*_^*2*^ + 7.301 × *R*_*Skewness*_^3^	Polynomial fitting

F_1_: Using the mean parameters R_Mean_, G_Mean_, B_Mean_ and their combinations with a normality assumption to establish multivariate linear regression models by stepwise regression, then choosing the best model. F_2_: Using all 20 parameters to establish multivariate linear regression models by stepwise regression, then choosing the best model. F_3_: Using the Fourier function to fit and obtain the model. F_4_: Using the MATLAB Curve Fitting Toolbox to fit the polynomial F4 that incorporates spatial bidirectional patterns

In order to assess the advantages and disadvantages of the four models, we compare their fitting performance (Table 5). The models F_2_, F_3_ and F_4_ had higher R^2^. The model F_4_ increased 21% compared with the model F_1_. To evaluate the prediction accuracy of the four models, we collected another batch of leaf images with four values of leaf ages and 50 blades for each age value (Table 5). The models F_2_ and F_4_ had more accurate prediction, and the accuracy of F_4_ increased 5% compared with F_1_. The SSE and RMSE metrics of the F_4_ model were superior to those of the other models. Therefore, the model F_4_ based on the spatial feature polynomial with the spatial bidirectional patterns is the optimal model.

**Table 5:** Correlation between the leaf color parameters and the SPAD values for each of the constructed models

Model	R^2^	Adjusted R^2^	SSE	RMSE	Prediction sample	Eliminate abnormal	Predictive accuracy (%)	Standard deviation
F_1_	0.583	0.581	7168	6.017	168	8	78.17	0.1832
F_2_	0.694	0.689	5260	5.181	168	16	79.36	0.1976
F_3_	0.648	0.643	6048	5.555	168	13	64.42	0.2320
F_4_	0.719	0.705	4870	5.050	168	11	82.15	0.1732

The R^2^, adjusted R^2^, SSE, RMSE, predictive accuracy and standard deviation were compared for the four models (F_1_ − F_4_). Predictive accuracy = (1 − | predictive value-measured value |/measured) × 100%

## Discussion

In the past, the use of the RGB models for leaf color analysis had obvious limitations. The biggest drawback of such model was that it had too few parameters to use, only the mean values of the red, green, blue, and grayscale intensities [24]. Although previous studies have proposed a variety of models based on combinations of these parameters, no plausible explanation was given for the physiological significance of these parameters in describing leaf color changes [21, 22]. The reason for this was that when RGB features were extracted from digital images, the descriptive statistics were based on a normal distribution. This normality assumption is only a convenience for finding approximate values, but it cannot reflect the distribution of leaf colors in a comprehensive and truthful way.

In this work, we verified through general normality tests that the RGB color gradation histogram followed a skewed distribution for tobacco leaves with different leaf ages. As a result, we extend the color gradation distribution parameters in the RGB model. These parameters include the mean, median, mode, skewness, and kurtosis. This gives a total of 20 parameters for 4 channels, while the common normal-distribution parameter is only the mean value.

Each of these parameters reflects some property or trait of leaf color. When the mean value is extracted based on a normality assumption, the leaf color heterogeneity is ignored. The mean can only describe the state of the leaf color depth quantitatively. This cannot fully reflect a real leaf color distribution at any leaf age. The description of the skewed distribution not only expands quantitative leaf color information but also systematically characterizes the leaf color depth and homogeneity. The skewness and kurtosis are features that mainly reflect the leaf color homogeneity. These features make it possible to accurately and quantitatively describe leaf color from different aspects.

We found 17 of the 20 parameters to be significantly correlated with the SPAD value at the 0.01 significance level. We try to model the chlorophyll content and distribution of leaves with these parameters. In earlier studies, the mean parameters of the R, G, and B components as well as their combinations were generally used with a normality assumption to establish models by stepwise regression. We also used this method to get the model F_1_. After comparing the models F_2_, F_3_ and F_4_ with F_1_ using skewed-distribution parameters, we found that the model based on the median and the skewness could better fit the SPAD value. More parameters increased the accuracy of the RGB model description and prediction, and extended its application range. When we used the Fourier method in the model F_3_, we found that the fitting degree was higher than that in the model F_1_, indicating that the numerical SPAD distribution was more in line with the curve distribution. Predicting the SPAD value with the mean value only didn’t work well. This means that the depth of the leaf color cannot describe the leaf color accurately. When introduced the skewness, and found that both the fitting degree and the prediction accuracy were greatly improved. So, these skewed-distribution parameters can describe changes in leaf color depth and homogeneity.

To sum up, the color distribution histogram of blade images follows a skewed distribution, whose parameters (such as the mean, median, mode, skewness, and kurtosis) greatly enrich the RGB model. We hope that this work will provide researchers with a new method for the analysis of blade color patterns in RGB digital images. This work shall also inspire the extraction and exploitation of novel leaf color descriptors for plant monitoring and treatment.

## Data Availability

The datasets used and/or analysed during the current study are available from the corresponding author on reasonable request.
